# A novel *ANK1* gene mutation associated with hereditary spherocytosis: a case report

**DOI:** 10.3389/fped.2026.1760131

**Published:** 2026-05-29

**Authors:** Mingqian Lai, Zhengqiang Luo, Zhenyu Yang, Ronghua Pan, Weijun Huang, Wensen Zhang, Guoda Ma, Riling Chen

**Affiliations:** 1 Department of Pediatric Hematology and Oncology, Shunde Women and Children’s Hospital (Maternity and Child Healthcare Hospital of Shunde Foshan), Guangdong Medical University, Foshan, Guangdong, China; 2 Department of Pediatrics, Shunde Women and Children’s Hospital (Maternity and Child Healthcare Hospital of Shunde Foshan), Guangdong Medical University, Foshan, Guangdong, China; 3 Maternal and Child Research Institute, Shunde Women and Children’s Hospital (Maternity and Child Healthcare Hospital of Shunde Foshan), Guangdong Medical University, Foshan, Guangdong, China

**Keywords:** hereditary spherocytosis, ANK1 mutation, splice-site variant, next-generation sequencing, splenectomy

## Abstract

**Background:**

Hereditary spherocytosis (HS) is an inherited form of hemolytic anemia resulting from defects in the red blood cell membrane skeleton. Its classic clinical presentation includes anemia, jaundice, and splenomegaly. Key laboratory findings that support the diagnosis are the presence of spherocytes and an elevated reticulocyte count on peripheral blood smear.

**Case presentation:**

An 8-year-old girl presented with a longstanding history of jaundice—noted for four years—affecting both her skin and sclera. She also reported intermittent episodes of tea-colored urine. Physical examination revealed hepatosplenomegaly. Laboratory investigations demonstrated normocytic anemia, a positive osmotic fragility test, and the presence of spherocytes on peripheral blood smear, which was subsequently confirmed by electron microscopy. Whole-exome sequencing identified a novel heterozygous pathogenic mutation (c.2388 + 2T > A) in the *ANK1* gene (NM_000037.4, Intron). This splice-site mutation leads to aberrant splicing, causing a frameshift and introduction of a premature termination codon (PTC), likely triggering nonsense-mediated mRNA decay (NMD) and resulting in a truncated, dysfunctional Ankyrin-1 protein.

**Conclusion:**

This study reports a novel *ANK1* mutation (c.2388 + 2T > A) identified in a Chinese patient with hereditary spherocytosis. Located at a critical splice donor site, this previously unreported variant is predicted to cause disease through the inclusion of a cryptic exon. The finding provides new insight into the genetic basis of HS in the Chinese population.

## Background

Hereditary spherocytosis (HS) is an inherited hemolytic anemia caused by defects in red blood cell membrane skeletal proteins. The condition manifests clinically with anemia, jaundice, and splenomegaly. Laboratory diagnosis typically relies on the peripheral blood smear, which shows increased numbers of spherocytes and reticulocytes (1). In the Chinese population, the reported prevalence of HS is approximately 1.27 and 1.49 per 100,000 in males and females, respectively (2). The underlying genetic causes are well-established, involving mutations in five major genes that encode key membrane proteins: *SPTA1* (*α*-spectrin), *SPTB* (*β*-spectrin), *ANK1* (ankyrin-1), *SLC4A1* (band 3), and *EPB42* (protein 4.2) (3). Among these, mutations in the *ANK1* gene are the most common, accounting for roughly half of all HS cases (4).

## Case presentation

### Baseline information

An 8-year-old girl presented to our hospital with a four-year history of recurrent jaundice and intermittent passage of tea-colored urine. The condition began insidiously four years ago with persistent scleral and skin discoloration. Throughout its course, she has experienced episodes of dark urine, typically in the mornings, without associated constitutional symptoms. She was previously hospitalized for hyperbilirubinemia, during which the dark urine resolved with treatment, but the jaundice persisted. Following discharge, the morning episodes of tea-colored urine recurred, leading to the current admission for further evaluation. On admission, her vital signs were stable: blood pressure 102/65 mmHg, heart rate 96 beats per minute, respiratory rate 22 breaths per minute, and oxygen saturation 98% on room air. Physical examination revealed prominent icterus involving the sclera, facial skin, and chest wall, along with marked pallor of the lips and face. Abdominal examination revealed a flat contour without tenderness or guarding. The liver edge was palpable 3 cm below the right costal margin; it was soft and smooth with no palpable nodules. The spleen was palpable 6 cm below the left costal margin, firm, well-defined, and non-tender. She is the only child, and there is no known family history of jaundice, anemia, or hematologic disorders.

### Diagnostic investigations

**Figure 1:**
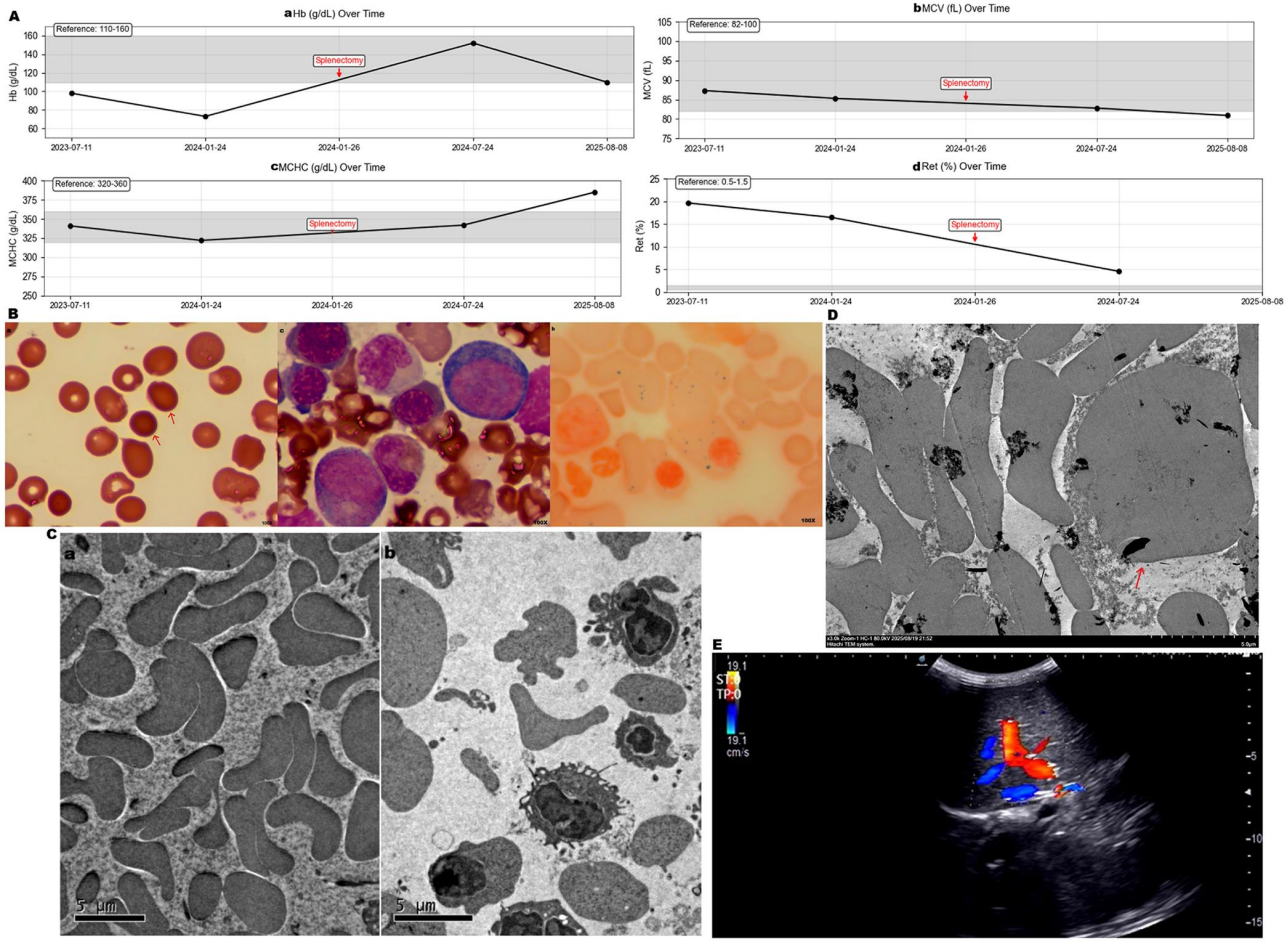
Diagnostic findings **(A).** Perioperative complete blood count trends, with arrow indicating splenectomy timing. **(B) (a)** Peripheral blood smear shows ∼20% spherocytes (arrows), characterized by loss of central pallor, with mild anisocytosis and microcytosis. **(b)** Iron staining demonstrates normal intracellular iron storage, evidenced by preserved central pallor in sideroblasts. **(c)** Bone marrow aspirate exhibits morphology consistent with hemolytic anemia. **(C)** Electron micrograph of bone marrow shows mature erythrocytes with scattered erythroid precursors and leukocytes. Erythroblasts display irregular morphology with mildly dilated perinuclear spaces. A minor proportion (<5%) of erythrocytes appear as spherocytes with size variation. **(D)** Representative field shows mature erythrocytes with occasional spherocytes (arrows). **(E)** Abdominal ultrasonography demonstrates mild hepatosplenomegaly: right liver lobe 10.5 cm (reference: ∼10.0 cm) with 2.8 cm subcostal extension; spleen thickness 3.7 cm (reference: ∼3.5 cm) with homogeneous echogenicity.

A comprehensive diagnostic workup was performed to determine the etiology of the anemia. The investigations included an erythrocyte osmotic fragility test, complete blood count (Figure 1A), liver function tests, bone marrow and peripheral blood cytomorphology (Figure 1B), electron microscopy of bone marrow and peripheral blood for ultrastructural examination (Figures 1C,D), an iron metabolism panel, a direct antiglobulin (Coombs) test, flow cytometry for PNH clones, thalassemia genetic testing, a glucose-6-phosphate dehydrogenase (G6PD) activity assay, and hepatobiliary-splenic ultrasonography (Figure 1E). Laboratory findings were consistent with a compensated hemolytic anemia, characterized by a markedly elevated reticulocyte count and increased erythrocyte osmotic fragility. The peripheral blood smear showed a spherocyte proportion exceeding 10%. Bone marrow morphology further supported the diagnosis of hemolytic anemia, while abdominal ultrasonography confirmed hepatosplenomegaly and showed no evidence of cholelithiasis. Electron microscopy identified small numbers of typical spherocytes in both bone marrow and peripheral blood specimens. Taken together, these results effectively ruled out other common causes of anemia and confirmed the clinical diagnosis of hereditary spherocytosis.

### Genetic testing

**Figure 2:**
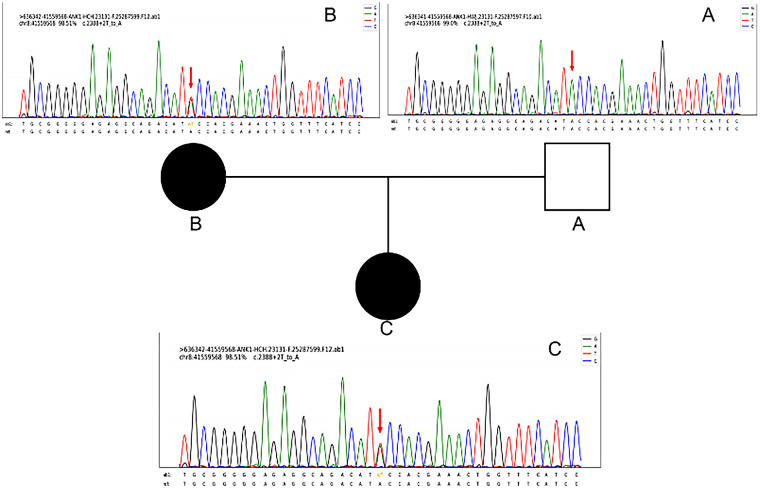
Sanger sequencing confirmed the heterozygous *ANK1* c.2388 + 2T > A mutation in the proband and her mother. Squares and circles represent male and female individuals, respectively; filled symbols indicate mutation carriers. The red arrow indicates the variant position. **(A)** Father (wild-type). **(B)** Mother (heterozygous mutant). **(C)** Patient (heterozygous mutant).

After obtaining informed consent from the guardians, peripheral blood samples (3 mL each, EDTA-anticoagulated) were collected from the patient and her parents. Next-generation sequencing was performed by Guangzhou Jiajian Medical Testing, targeting the exonic regions and clinically relevant intronic variants of 5,595 disease-associated genes curated from the OMIM database. Analysis identified a heterozygous mutation (c.2388 + 2T > A) in intron 21 of the *ANK1* gene (NM_000037.4) in the patient (Figure 2). Although initially classified as a variant of uncertain significance, subsequent functional validation (see below) provided evidence for reclassification. The mutation affects the highly conserved ‘GT’ dinucleotide of the canonical splice donor site. Interrogation of major public databases (gnomAD, VarCards, HGMD) confirmed that this variant is novel, with no prior population or disease-associated records.

### Diagnosis and treatment

The patient presented with the classic triad of hemolytic anemia: anemia, jaundice, and hepatosplenomegaly. Laboratory confirmation included the presence of spherocytes on peripheral blood smear and bone marrow electron microscopy, elevated reticulocyte count, and increased erythrocyte osmotic fragility. Genetic testing identified a pathogenic mutation in the *ANK1* gene. Following the exclusion of alternative diagnoses—including thalassemia, autoimmune hemolytic anemia, G6PD deficiency, paroxysmal nocturnal hemoglobinuria, and viral hepatitis—a definitive diagnosis of hereditary spherocytosis was established. As the patient met the established criteria for splenectomy (5), surgical intervention was recommended. Preoperatively, she received essential vaccinations to reduce the risk of post-splenectomy infections. According to guidelines, she was vaccinated against encapsulated bacteria at least 2 weeks before surgery, including pneumococcal (PCV13 and PPSV23), meningococcal (quadrivalent and serogroup B), and Haemophilus influenzae type b (Hib) vaccines. In addition, she received oral folic acid 5 mg/day for 4 weeks before surgery. A fluorescence-guided laparoscopic partial splenectomy, which included resection of an accessory spleen, was successfully performed on January 26, 2024. Postoperatively, she continued folic acid supplementation for 3 months. No surgical complications occurred. Follow-up at 3 months showed hemoglobin 118 g/L, reticulocytes 3.2%, and resolution of jaundice. No further episodes of tea-colored urine were reported, and the patient had returned to school with improved energy.

### Pathogenicity assessment

**Figure 3:**
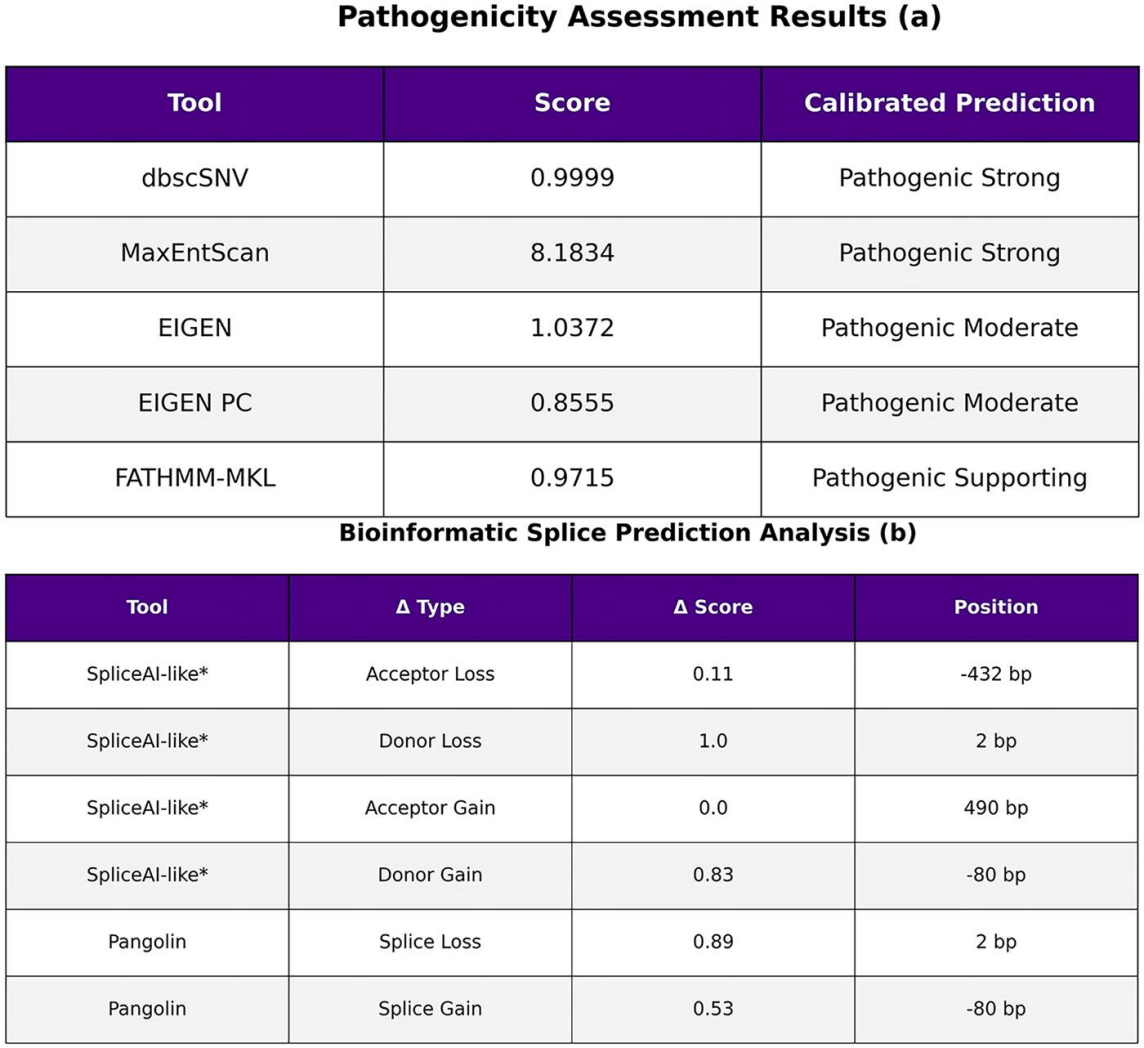
Pathogenicity assessment of the *ANK1* c.2388 + 2T > A variant. **(a)** multiple computational algorithms consistently support a deleterious effect of the variant, with high-confidence pathogenic predictions across independent tools. **(b)** Both SpliceAI and Pangolin algorithms predict disruption of the canonical donor splice site with concurrent activation of a cryptic donor site, indicating a high probability of aberrant splicing.

To evaluate the pathogenicity of the identified *ANK1* variant (c.2388 + 2T > A), an *in silico* analysis was performed using the VarSome platform (https://varsome.com/). The variant is located at the highly conserved canonical splice donor site, indicating a likely disruption of normal mRNA splicing and subsequent protein dysfunction. Multiple bioinformatic tools yielded convergent evidence supporting its deleterious nature (Figure 3a). The dbscSNV and MaxEntScan algorithms strongly predicted a damaging effect on splicing. Both the EIGEN and EIGEN PC scores suggested moderate pathogenicity, and the FATHMM-MKL algorithm also indicated a deleterious outcome. These consistent computational predictions strongly support the pathogenic potential of this novel *ANK1* splice-site variant.

### Splice effect prediction

To evaluate the potential effect of this variant on mRNA splicing, we conducted computational predictions using the SpliceAI online tool (https://spliceailookup.broadinstitute.org). The analysis indicated two potential splicing alterations: a donor loss at the 2 bp downstream position (score = 1.00) and the acquisition of a new acceptor site approximately 80 bp upstream (score = 0.83). Subsequent evaluation with the Pangolin tool corroborated these findings, revealing both splice loss (score = 0.89) and splice gain (score = 0.53) events (Figure 3b), demonstrating strong concordance with the SpliceAI predictions. Together, these computational analyses suggest that the c.2388 + 2T > A variant is likely to disrupt the canonical donor splice site, potentially activating a cryptic upstream splice donor and leading to abnormal mRNA splicing.

### Experimental validation

**Figure 4:**
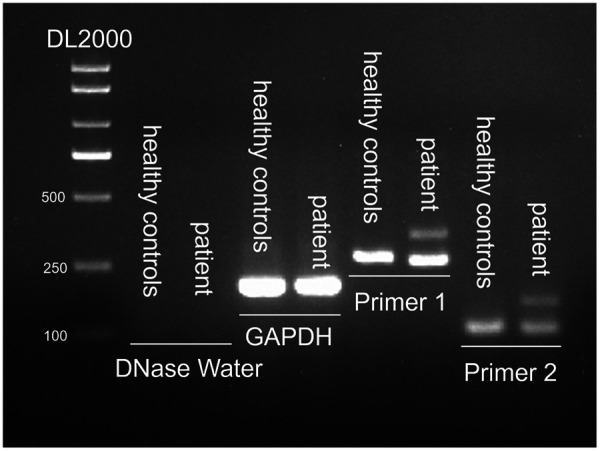
Splicing Analysis of the *ANK1* c.2388 + 2T > A Variant. Agarose gel electrophoresis of cDNA PCR products demonstrates an abnormally larger amplicon in the proband, consistent with an approximately 50 bp insertion in the *ANK1* transcript, thus confirming the variant's role in disrupting canonical splicing.

To investigate the effect of the *ANK1* c.2388 + 2T > A mutation on mRNA splicing, total RNA was extracted from the peripheral blood of the patient and a wild-type control, followed by reverse transcription to cDNA. PCR amplification was performed using two exon-spanning primer sets: set 1 (F: 5'-AAGCTAGGATACAGCCCCCT-3’, R: 5'-AGTTCTTCCCCTTCATCTTCCG-3’) spanning exons 20–22, and set 2 (F: 5'-CTGAAAAACGGTGCTTCCCC-3’, R: 5'-GGTTTCATCCGTGACGACCT-3’) spanning exons 21–22. GAPDH (F: 5'-ACGGCAAATTCAACGGCACAG-3’, R: 5'-ACACCAGTAGACTCCACGACATAC-3’) was co-amplified as an internal control, and nuclease-free water served as a no-template control to ensure specificity. Agarose gel (1%) electrophoresis of the PCR products revealed a consistent abnormal finding: the patient’s samples produced an additional band approximately 50 bp larger than the wild-type control with both primer sets. All control reactions yielded the expected results, confirming amplification specificity. These results demonstrate that the patient carries a heterozygous mutation leading to the insertion of an additional nucleotide sequence. Based on the primer design, the insertion is localized between exons 21 and 22. This finding is consistent with the inclusion of a cryptic exon, likely resulting from the activation of a cryptic splice donor site as predicted by bioinformatic analysis (Figure 4). According to ACMG/AMP guidelines, this variant meets PS3 (functional evidence of aberrant splicing by RT-PCR) and PM2 (absent from population databases). Therefore, it is classified as Pathogenic (Class 5).

## Patient perspective

The patient's mother shared her experience: “For four years, we were worried about my daughter's yellow color and dark urine, but no one could tell us the cause. She was unable to attend school normally and her physical development was poor. Since no one else in our family had similar symptoms, we had no clue what was happening. The genetic test finally gave us an answer. After the surgery, her color returned to normal, and she has much more energy. She is now back to school and growing better. We are grateful for the diagnosis and wish other families could get genetic testing earlier.”

## Discussion

This study identified a novel heterozygous *ANK1* mutation (c.2388 + 2T > A) with maternal inheritance. Hereditary spherocytosis (HS) follows an autosomal dominant pattern in approximately 75% of cases, predominantly due to heterozygous *ANK1* mutations (6). Although clinical features are often consistent within a family, significant variation in disease severity between different families is well recognized (5). Our pedigree clearly illustrates this complexity and highlights notable intrafamilial heterogeneity. The proband's mother carries the same pathogenic variant. Laboratory studies revealed evidence of compensated hemolysis—including 18% microspherocytes on peripheral smear, a hemoglobin level of 117 g/L, and elevated indirect bilirubin (total bilirubin 45.29 μmol/L)—yet she remains entirely asymptomatic, consistent with a mild or subclinical form of HS. In stark contrast, her daughter, who carries the identical mutation, presented with overt hemolytic symptoms such as recurrent jaundice and tea-colored urine, ultimately requiring splenectomy. This marked phenotypic disparity underscores the poor correlation between genotype and clinical phenotype often observed in HS (7). The heterogeneity may be influenced by several factors, including modifier genes (e.g., *UGT1A1* involved in bilirubin metabolism) and individual differences in bone marrow compensatory capacity (8). A well-characterized modifier in HS is *UGT1A1*, which encodes the enzyme responsible for bilirubin conjugation. Common polymorphisms in the *UGT1A1* promoter reduce enzymatic activity, leading to unconjugated hyperbilirubinemia. In the setting of chronic hemolysis, this defect can significantly aggravate jaundice and increase the risk of cholelithiasis (9). Potential modifiers, including variants in genes involved in bilirubin metabolism such as UGT1A1 or concurrent polymorphisms in other membrane-stabilizing proteins, may further mitigate or exacerbate the clinical expression of the ANK1 defect. Consequently, personalized clinical evaluation, rather than reliance on genotypic information alone, should be the cornerstone of management and genetic counseling for HS families.

Experimental validation confirmed that the *ANK1* c.2388 + 2T > A mutation consistently generated an abnormal PCR product approximately 50–60 bp larger than the wild-type control. This size discrepancy is directly attributed to the mutation's disruption of the canonical splice donor site and the concomitant activation of a cryptic upstream donor, leading to the mis-splicing and inclusion of a 50–60 bp intronic segment as a pseudoexon. This aligns with a recognized pathogenic mechanism wherein deep intronic mutations creating novel splice sites frequently cause the aberrant incorporation of intronic sequence into the mature transcript (10). Such aberrant splicing typically introduces premature termination codons, thereby triggering nonsense-mediated mRNA decay (NMD). The consequent loss of functional Ankyrin-1—the critical structural protein encoded by *ANK1* that maintains erythrocyte membrane stability—ultimately underlies the disease pathogenesis.

Erythrocyte Ankyrin-1 is a critical membrane skeletal protein that anchors the spectrin-based cytoskeleton to the lipid bilayer via interactions with *β*-spectrin and band 3, thereby maintaining membrane stability (11). Structurally, it comprises three principal domains: an N-terminal membrane-binding domain (with 24 tandem ankyrin repeats), a central spectrin/band 3-binding domain, and a less conserved C-terminal regulatory domain (12). The *ANK1* gene, located at chromosome 8p11.21, spans 49 exons (13). Exons 1–22 encode the crucial N-terminal domain (14, 15). In this patient, the c.2388 + 2T > A mutation results in the insertion of an aberrant sequence between exons 21 and 22. This insertion is predicted to induce nonsense-mediated mRNA decay (NMD), which would abolish the function of the N-terminal domain. This functional loss disrupts the critical linkage between the lipid bilayer and the spectrin cytoskeleton. Consequently, erythrocytes lose membrane surface area and deformability, assuming a rigid spherical shape. These fragile spherocytes are subsequently trapped and destroyed in the spleen, culminating in the hemolytic anemia that defines hereditary spherocytosis.

The diagnosis of hereditary spherocytosis (HS) typically relies on a combination of clinical evaluation, physical examination, peripheral blood smear analysis, and the osmotic fragility test. However, significant diagnostic challenges can arise due to variable clinical presentations—ranging from asymptomatic carriage to life-threatening anemia—and ambiguous family histories (16). Established laboratory methods, such as the osmotic fragility test and the eosin-5′-maleimide (EMA) binding flow cytometry assay, are valuable but have notable limitations. The osmotic fragility test lacks optimal sensitivity and specificity, especially in mild or recently transfused cases. While the EMA binding assay offers greater objectivity, it remains susceptible to technical artifacts and sample hemolysis (17). Consequently, achieving an accurate diagnosis often requires a multifaceted approach. The integration of molecular techniques is increasingly crucial. With the decreasing cost of next-generation sequencing (NGS), this technology is now routinely applied in the genetic diagnosis of inherited hemolytic anemias, including HS. NGS allows for the high-throughput analysis of known pathogenic genes (e.g., *ANK1*, *SPTB*), providing definitive evidence for diagnosis and facilitating differential diagnosis (18).

## Limitations

Several limitations should be acknowledged. First, this is a single case report, which limits generalizability. Second, we did not perform protein-level validation due to lack of available antibodies. Third, UGT1A1 genotyping was not performed to assess its potential modifier effect on the mother's mild phenotype. Despite these limitations, the RT-PCR functional validation provides strong evidence for pathogenicity.

## Conclusion

In conclusion, this study identifies a novel pathogenic *ANK1* splice-site mutation (c.2388 + 2T > A) in a Chinese patient with hereditary spherocytosis. This discovery expands the known mutational spectrum of HS and underscores the critical role of next-generation sequencing in diagnosing atypical cases and elucidating the genetic architecture of this disorder.

## Data Availability

The original contributions presented in the study are included in the article/Supplementary Material, further inquiries can be directed to the corresponding author.
