# Supplementary material for: A novel ANK1 gene mutation associated with hereditary spherocytosis: a case report
Source: Front Pediatr. 2026 May 29;14:1760131. doi: 10.3389/fped.2026.1760131 (PMC13260293; doi:10.3389/fped.2026.1760131)
Supplement: Supplementary file 1 [file Datasheet1.pdf]

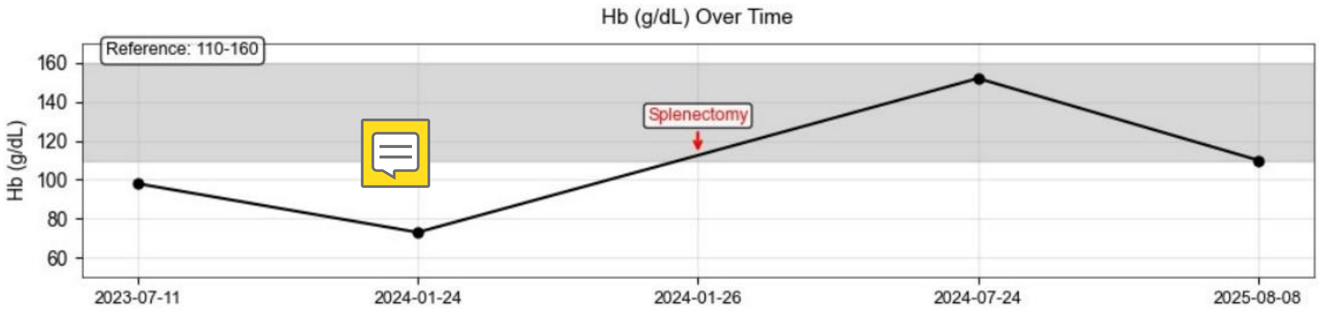
Hb (g/dL) Over Time
Reference: 110-160
Splenectomy
Hb (g/dL)
160
140
120
100
80
60
2023-07-11
2024-01-24
2024-01-26
2024-07-24
2025-08-08

figure-1-A-a

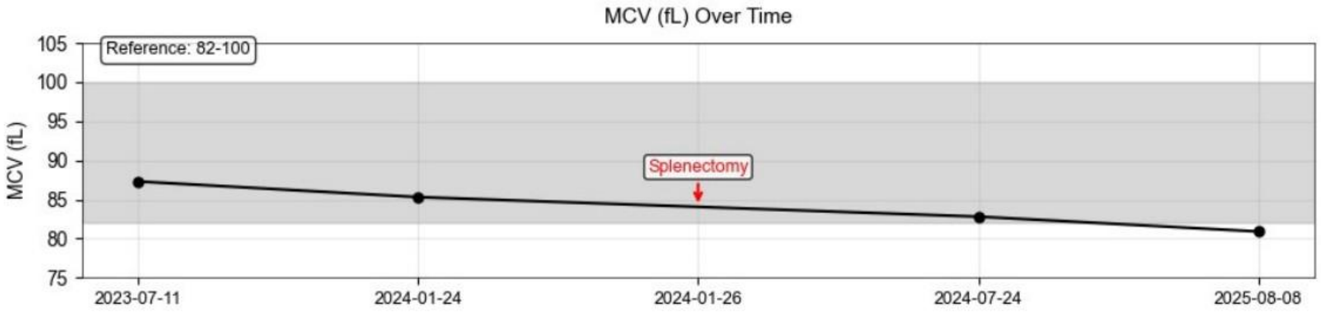
MCV (fL) Over Time
Reference: 82-100
Splenectomy
MCV (fL)
105
100
95
90
85
80
75
2023-07-11
2024-01-24
2024-01-26
2024-07-24
2025-08-08

figure-1-A-b

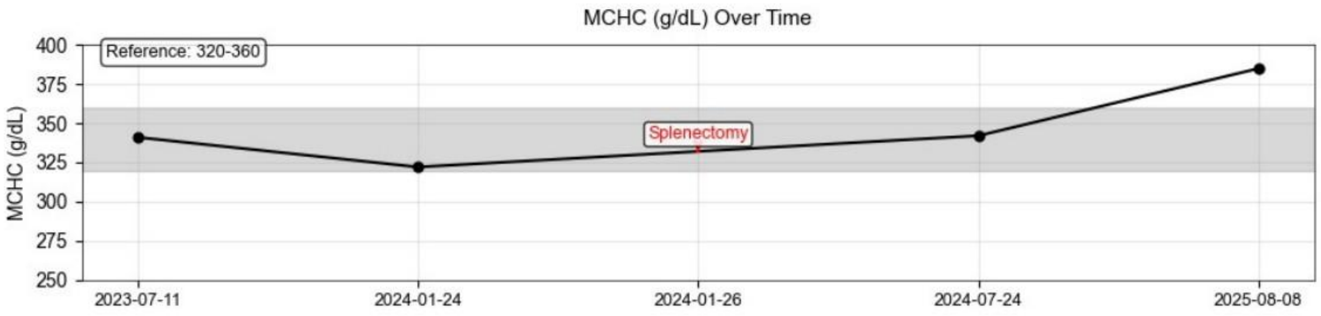
MCHC (g/dL) Over Time
Reference: 320-360
MCHC (g/dL)
400
375
350
325
300
275
250
Splenectomy
2023-07-11
2024-01-24
2024-01-26
2024-07-24
2025-08-08

figure-1-A-c

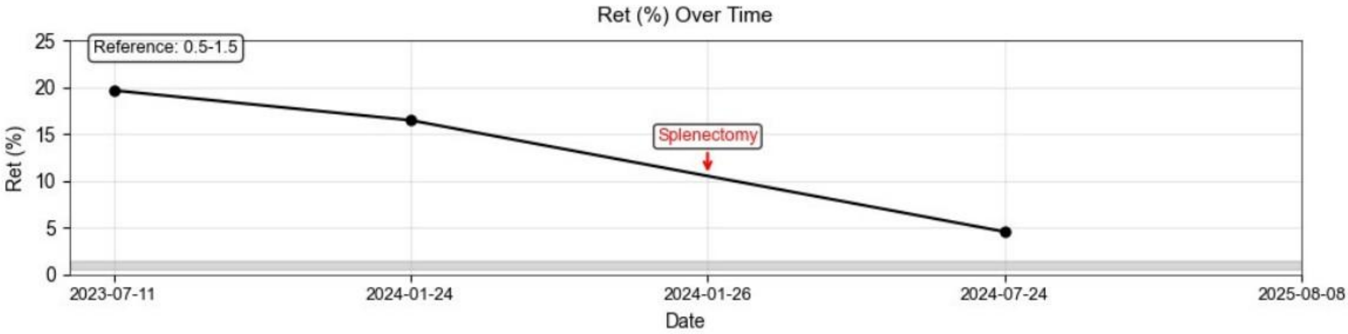
Ret (%) Over Time
Reference: 0.5-1.5
Splenectomy
Ret (%)
25
20
15
10
5
0
2023-07-11
2024-01-24
2024-01-26
2024-07-24
2025-08-08
Date

figure-1-A-d

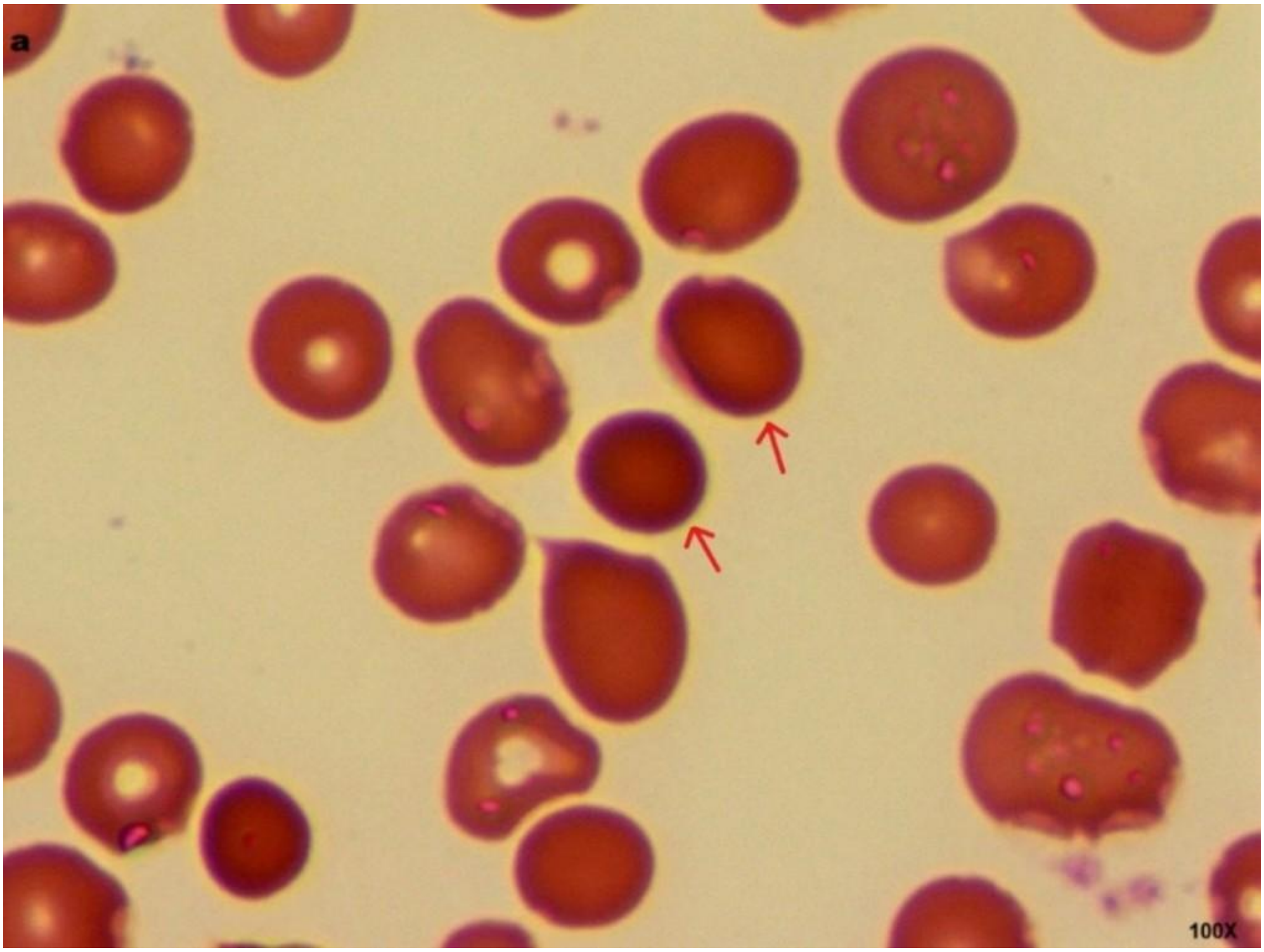

figure-1-B-a

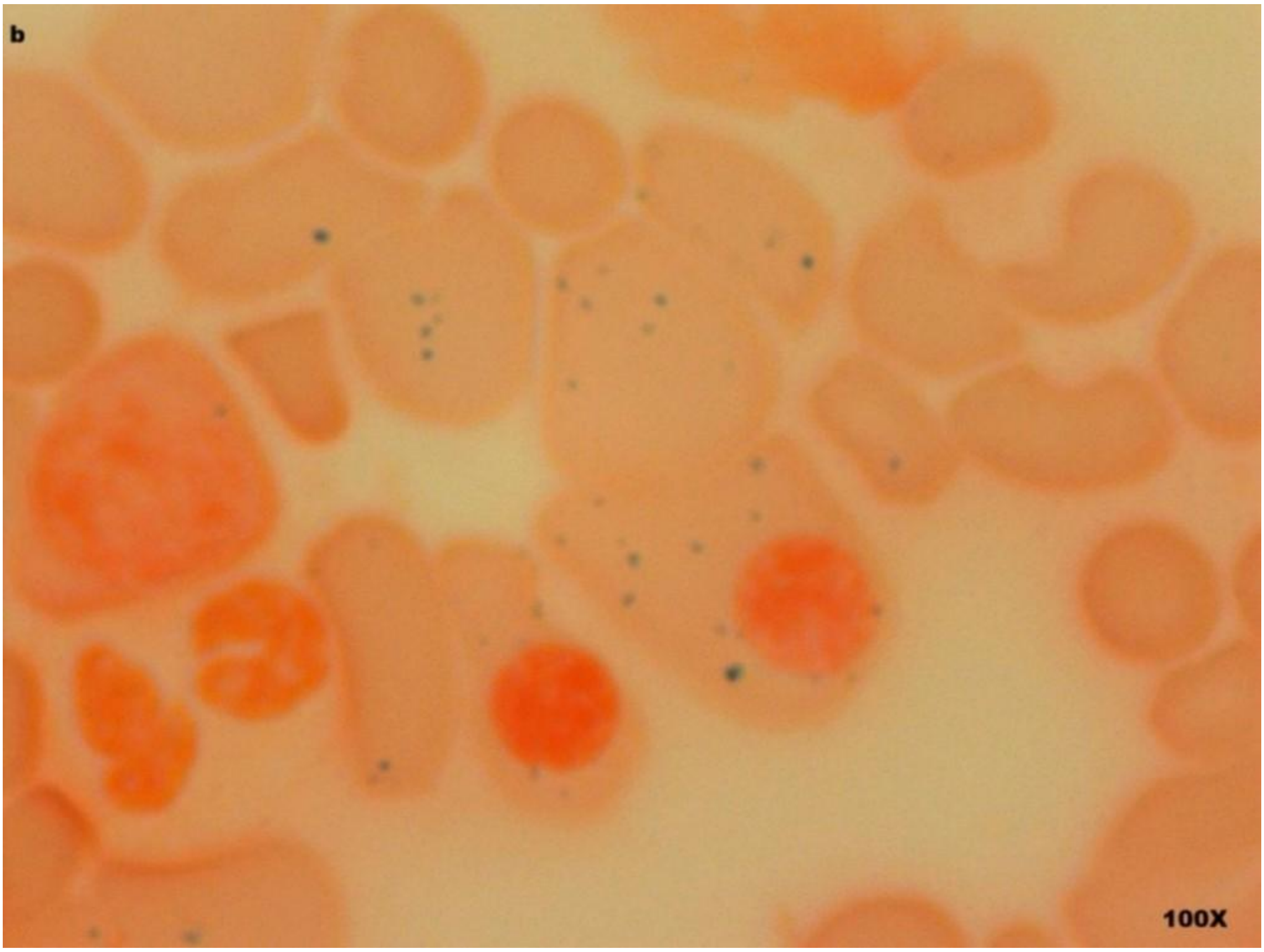

figure-1-B-b

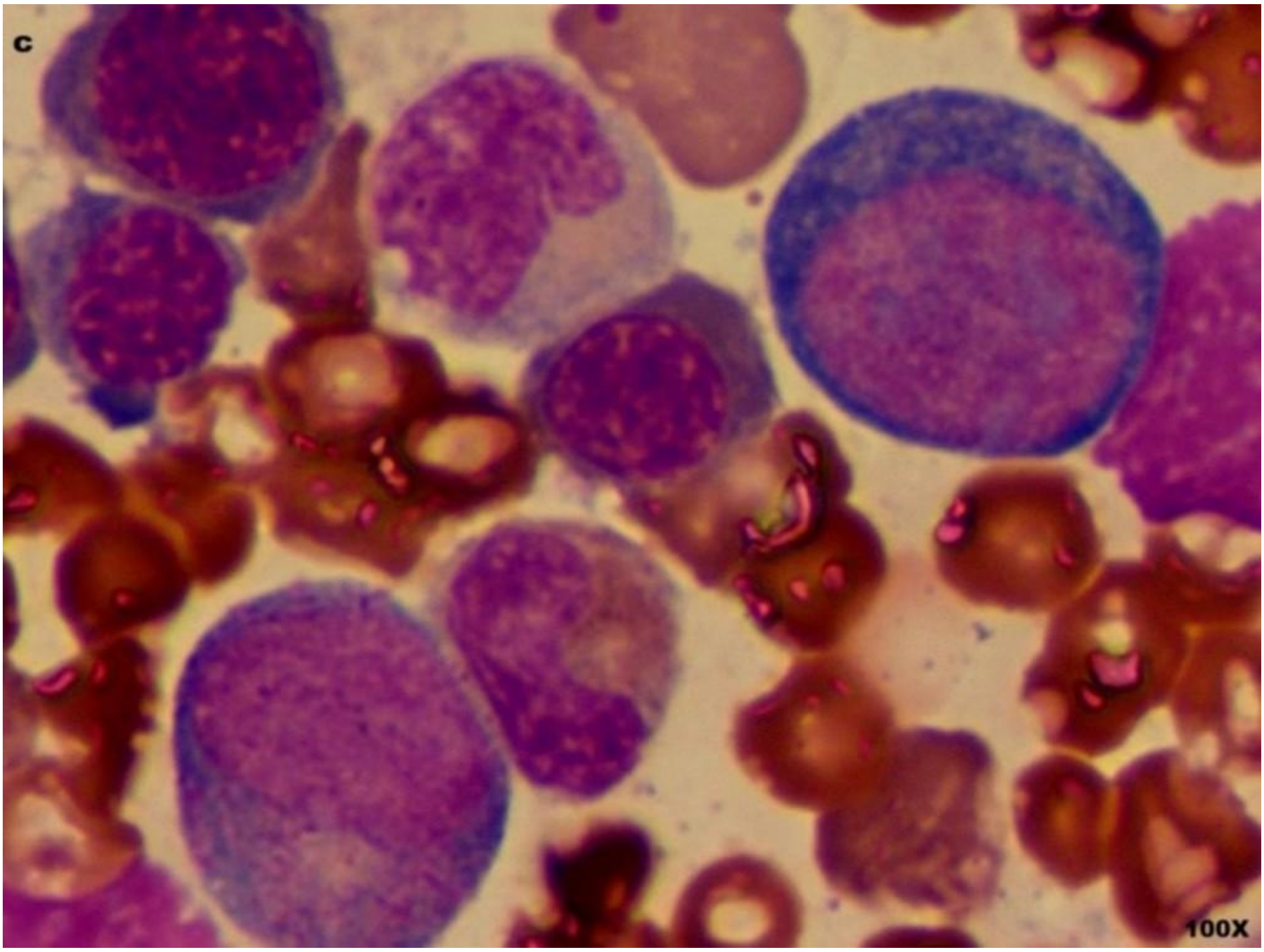

figure-1-B-c

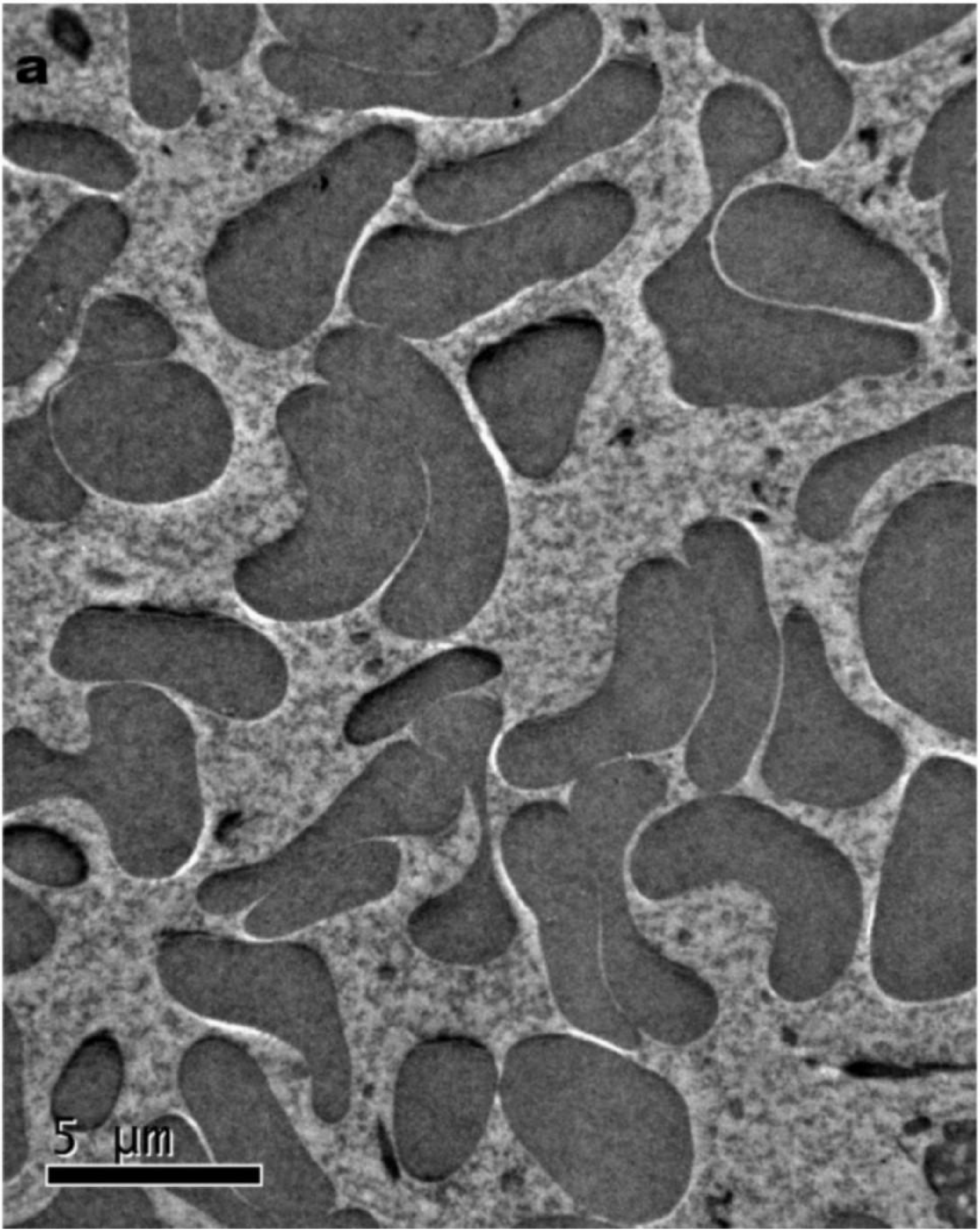

figure-1-C-a

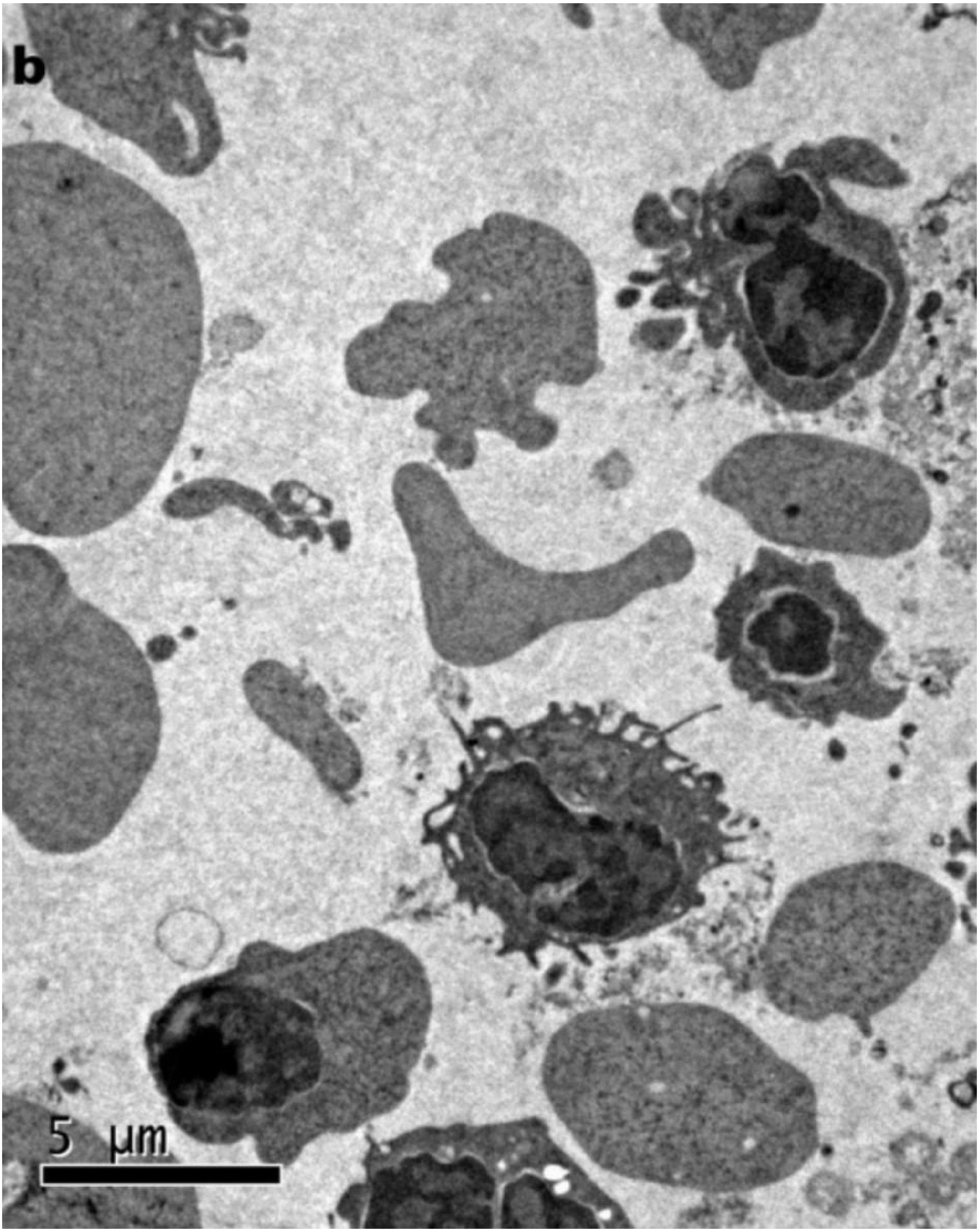

figure-1-B-b

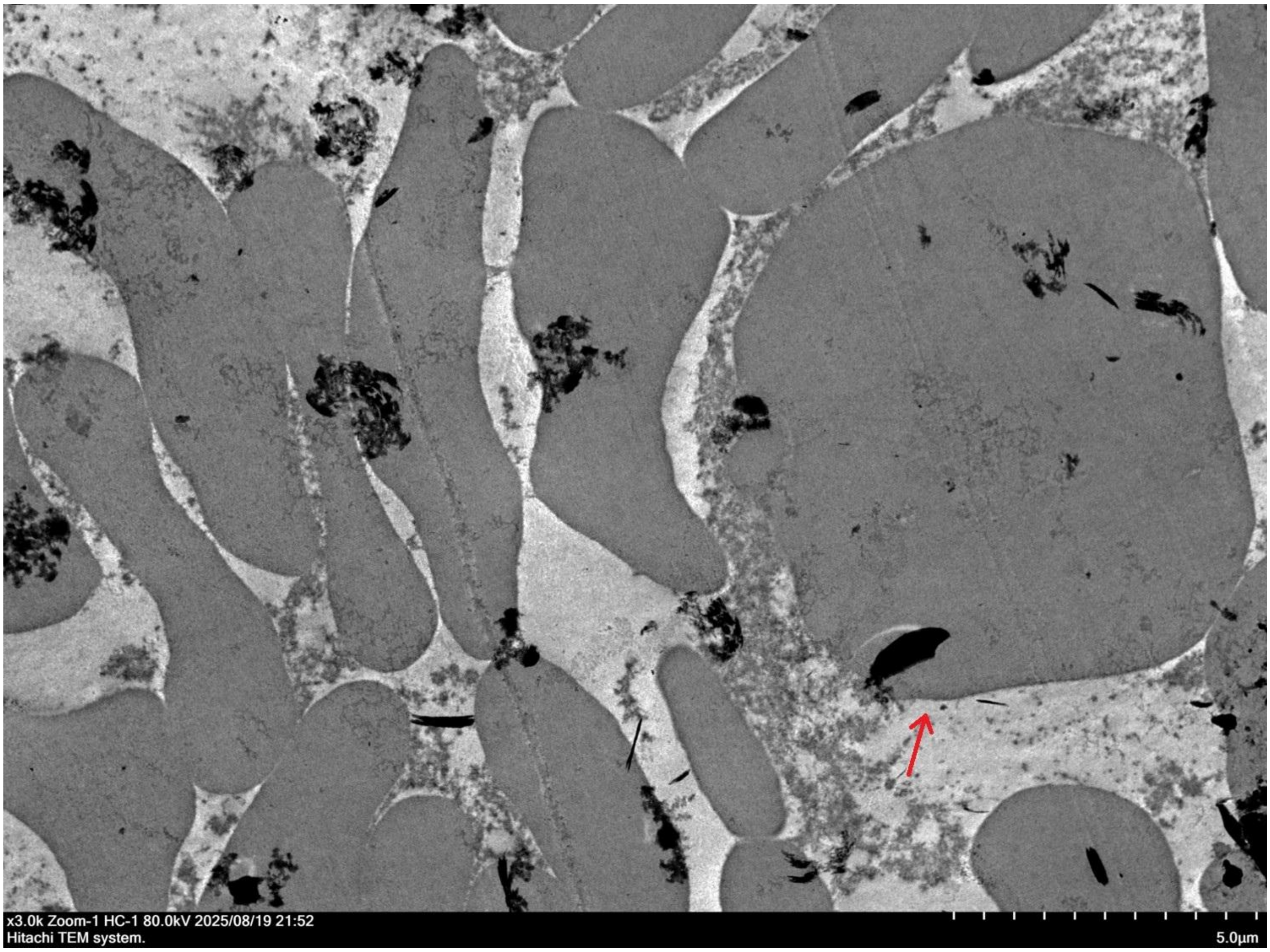

figure-1-D

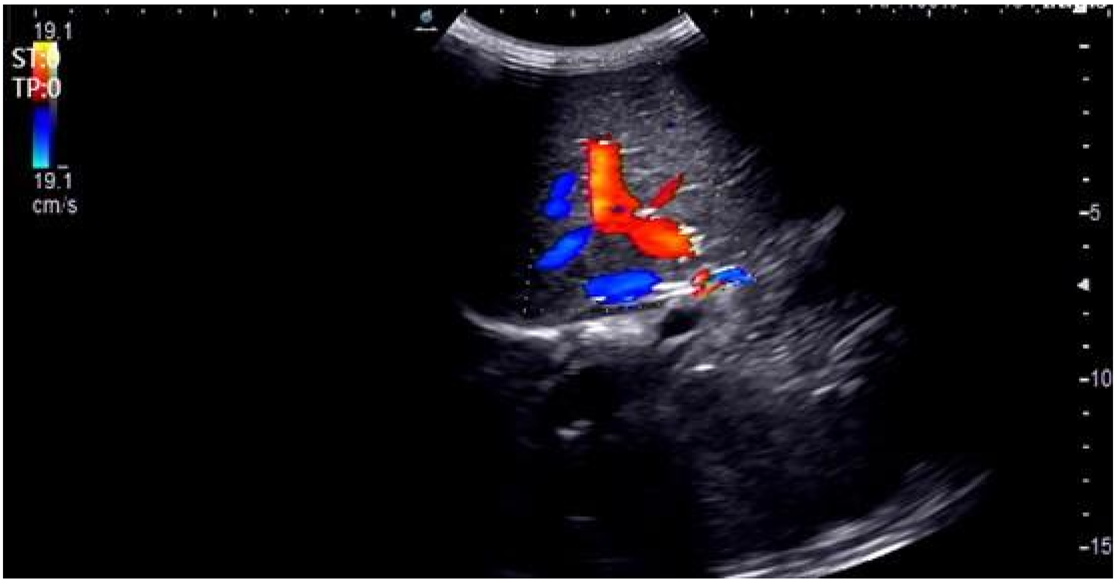

figure-1-E
