# Supplementary material for: A novel ANK1 gene mutation associated with hereditary spherocytosis: a case report
Source: Front Pediatr. 2026 May 29;14:1760131. doi: 10.3389/fped.2026.1760131 (PMC13260293; doi:10.3389/fped.2026.1760131)
Supplement: Supplementary file 2 [file Datasheet2.pdf]

Time point	Event
Age 4 years	First onset: jaundice, tea-colored urine
Age 7 years	Hospitalized for hyperbilirubinemia; jaundice persisted after treatment
Age 8 years (admission)	Physical exam: hepatosplenomegaly (liver 3 cm, spleen 6 cm);<br>hemoglobin 94 g/L, reticulocytes 12.5%, spherocytes >20%
Day 5 of admission	Whole-exome sequencing identified novel *ANK1* c.2388+2T>A
Day 12 of admission	RT-PCR confirmed aberrant splicing (cryptic exon insertion)
26-Jan-24	Laparoscopic partial splenectomy with accessory spleen resection
3 months post- splenectomy	Hemoglobin 118 g/L, reticulocytes 3.2%, jaundice resolved, no<br>tea- colored urine, returned to school
